# Crystal structure of 2,2,4-trimethyl-2,3,4,5-tetra­hydro-1*H*-benzo[*b*][1,4]diazepine hemihydrate

**DOI:** 10.1107/S2056989015013201

**Published:** 2015-07-15

**Authors:** K. S. Ezhilarasi, A. Akila, S. Ponnuswamy, B. K. Revathi, G. Usha

**Affiliations:** a PG and Research Department of Physics, Queen Mary’s College, Chennai-4, Tamilnadu, India; b PG and Research Department of Chemistry, Government Arts College, Coimbatore-18, Tamilnadu, India

**Keywords:** crystal structure, benzodiazepine, hydrogen bonding

## Abstract

The title compound, C_12_H_18_N_2_·0.5H_2_O, crystallizes with two independent organic mol­ecules (*A* and *B*) in the asymmetric unit, together with a water mol­ecule of crystallization. The diazepine rings in each mol­ecule have a chair conformation. The dihedral angle between benzene ring and the mean plane of the diazepine ring is 21.15 (12)° in mol­ecule *A* and 17.42 (11)° in mol­ecule *B*. In the crystal, mol­ecules are linked by N—H⋯O and O—H⋯N hydrogen bonds, forming zigzag chains propagating along [001].

## Related literature   

For examples of biological activities of benzodiazepines, see: De Baun *et al.* (1976 ▸). For the use of benzodiazepine derivatives as dyes for acrylic fibres, see: Harris & Straley (1968 ▸). For related structures, see: Thiruselvam *et al.* (2013 ▸); Lamkaddem *et al.* (2015 ▸); Ponnuswamy *et al.* (2006 ▸).
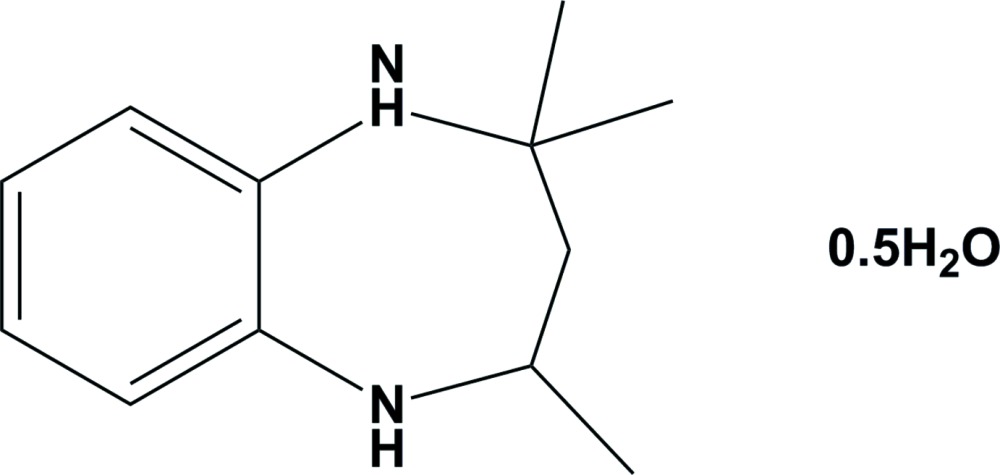


## Experimental   

### Crystal data  

C_12_H_18_N_2_·0.5H_2_O
*M* *_r_* = 199.29
Monoclinic, 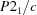

*a* = 9.0548 (10) Å
*b* = 23.246 (2) Å
*c* = 11.5613 (14) Å
β = 100.483 (3)°
*V* = 2392.9 (4) Å^3^
*Z* = 8
Mo *K*α radiation
μ = 0.07 mm^−1^
*T* = 293 K
0.30 × 0.25 × 0.20 mm

### Data collection  

Bruker Kappa APEXII CCD diffractometer
Absorption correction: multi-scan (*SADABS*; Bruker, 2008 ▸) *T*_min_ = 0.980, *T*_max_ = 0.986
22443 measured reflections
4161 independent reflections
2416 reflections with *I* > 2σ(*I*)
*R*_int_ = 0.042

### Refinement  

*R*[*F* ^2^ > 2σ(*F* ^2^)] = 0.051
*wR*(*F* ^2^) = 0.154
*S* = 1.03
4161 reflections
293 parameters
4 restraints
H atoms treated by a mixture of independent and constrained refinement
Δρ_max_ = 0.48 e Å^−3^
Δρ_min_ = −0.20 e Å^−3^

### 

Data collection: *APEX2* (Bruker, 2008 ▸); cell refinement: *SAINT* (Bruker, 2008 ▸); data reduction: *SAINT*; program(s) used to solve structure: *SHELXS97* (Sheldrick, 2008 ▸); program(s) used to refine structure: *SHELXL2014*/6 (Sheldrick, 2015 ▸); molecular graphics: *ORTEP-3 for Windows* (Farrugia, 2012 ▸) and *Mercury* (Macrae *et al.*, 2008 ▸); software used to prepare material for publication: *SHELXL2014*/6 and *PLATON* (Spek, 2009 ▸).

## Supplementary Material

Crystal structure: contains datablock(s) I, New_Global_Publ_Block. DOI: 10.1107/S2056989015013201/su5164sup1.cif

Structure factors: contains datablock(s) I. DOI: 10.1107/S2056989015013201/su5164Isup2.hkl

Supporting information file. DOI: 10.1107/S2056989015013201/su5164Isup3.cml

**Fig. 1.**
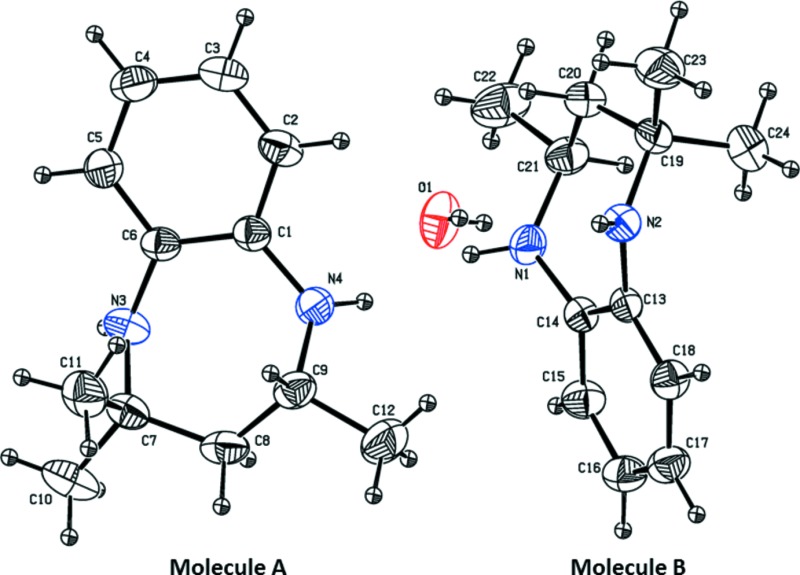
The molecular structure of the two independent molecules (A and B) of the title compound, with atom labelling. Displacement ellipsoids are drawn at the 30% probability level.

**Fig. 2.**
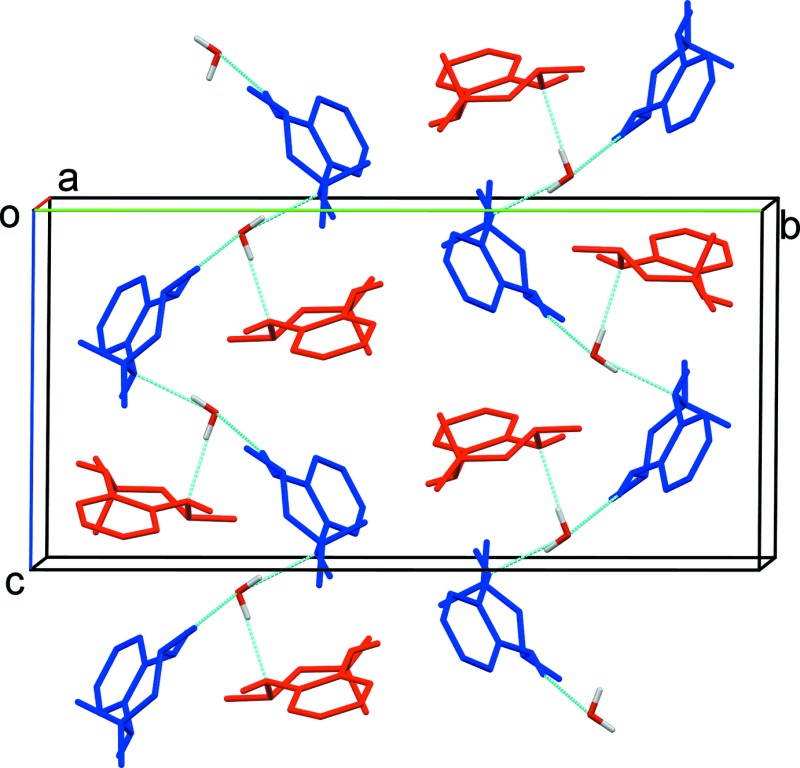
The crystal packing of the title compound, viewed along the a axis. The dashed lines indicate the hydrogen bonds (see Table 1 for details; molecule A red, molecule B blue).

. DOI: 10.1107/S2056989015013201/su5164fig1.tif
The mol­ecular structure of the two independent mol­ecules (A and B) of the title compound, with atom labelling. Displacement ellipsoids are drawn at the 30% probability level.

a . DOI: 10.1107/S2056989015013201/su5164fig2.tif
The crystal packing of the title compound, viewed along the *a* axis. The dashed lines indicate the hydrogen bonds (see Table 1 for details; mol­ecule A red, mol­ecule B blue).

CCDC reference: 1411680

Additional supporting information:  crystallographic information; 3D view; checkCIF report

## Figures and Tables

**Table 1:** Hydrogen-bond geometry (, )

*D*H*A*	*D*H	H*A*	*D* *A*	*D*H*A*
N1H1*N*O1^i^	0.84(2)	2.33(2)	3.122(3)	159(2)
O1H1*W*N4^ii^	0.84(4)	2.14(4)	2.976(4)	175(3)
O1H2*W*N2	0.86(4)	2.09(4)	2.930(3)	167(4)

Symmetry codes: (i) 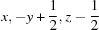; (ii) 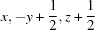.

## supplementary crystallographic information

### S1. Chemical context

Benzodiazepines have attracted attention as an important class of heterocyclic compounds in the field of drug design and pharmaceuticals. These compounds are widely used as anti­convulsant, anti-anxiety, analgesic, sedative, anti-depressive and hypnotic agents as well as anti-inflammatory agents (De Baun *et al.*, 1976). In addition to their potent biological activities, benzodiazepine derivatives are also used commercially as dyes for acrylic fibres (Harris & Straley, 1968).

### S2. Structural commentary

The title compound, crystallized with two independent organic molecules (*A* and *B*) in the asymmetric unit (Fig. 1). The C—C and C—N bond distances are normal and in good agreement with those reported for similar structures (Lamkaddem *et al.*, 2015; Ponnuswamy *et al.*, 2006; Thiruselvam *et al.*, 2013).

The diazepine rings each have a chair conformation. The dihedral angle between benzene ring and the mean plane of the diazepine ring is 21.15 (11)° in molecule *A* and and 17.42 (1)° in molecule *B*.

In the crystal of the title compound, molecules are linked through N—H···O and O—H···N hydrogen bonds, involving the water molecule, forming zigzag chains propagating along the *c* axis direction (Table 1 and Fig. 2).

### S3. Synthesis and crystallization

2,3-Di­hydro-2,2,4-tri­methyl-1H-tetra­hydro-1,5-benzodiazepine (9.10 mmol) was dissolved in methanol (40 ml) and stirred with a magnetic stirrer. Sodium borohydride (8.38 mmol) was added in three portions over a period of 1 h while maintaining the temperature at 318-323 K. After the addition was complete the solution was maintained at 323 K for 2 h. Methanol was evaporated partially and the reaction mass was poured into water and extracted with chloro­form several times. The organic extractions were combined, dried with anhydrous sulphate and then evaporated. The yellow oil obtained was purified by recrystallization from aqueous ethanol and afforded colourless crystals of the title compound (M.p.: 329-330 K).

### S4. Refinement

Crystal data, data collection and structure refinement details are summarized in Table 2. The NH and water H atoms were located in difference Fourier maps. The water H atoms were freely refined and the NH H atoms were refined with distance restraints; N–H = 0.86 (2) Å. The C-bound H atoms were positioned geometrically and treated as riding: C—H = 0.93-0.97 Å with *U*_iso_(H) = 1.5*U*_eq_(C) for methyl H atoms and 1.2U_eq_(C) for other H atoms.

### Crystal data

**Table:**

C_12_H_18_N_2_·0.5H_2_O	*F*(000) = 872
*M**_r_* = 199.29	*D*_x_ = 1.106 Mg m^−^^3^
Monoclinic, *P*2_1_/*c*	Mo *K*α radiation, λ = 0.71073 Å
*a* = 9.0548 (10) Å	θ = 1.8–24.9°
*b* = 23.246 (2) Å	µ = 0.07 mm^−^^1^
*c* = 11.5613 (14) Å	*T* = 293 K
β = 100.483 (3)°	Block, colorless
*V* = 2392.9 (4) Å^3^	0.30 × 0.25 × 0.20 mm
*Z* = 8	

### Data collection

**Table:**

Bruker Kappa APEXII CCD diffractometer	4161 independent reflections
Radiation source: fine-focus sealed tube	2416 reflections with *I* > 2σ(*I*)
Graphite monochromator	*R*_int_ = 0.042
ω and φ scan	θ_max_ = 24.9°, θ_min_ = 2.3°
Absorption correction: multi-scan (*SADABS*; Bruker, 2008)	*h* = −10→10
*T*_min_ = 0.980, *T*_max_ = 0.986	*k* = −27→26
22443 measured reflections	*l* = −13→13

### Refinement

**Table:**

Refinement on *F*^2^	Hydrogen site location: mixed
Least-squares matrix: full	H atoms treated by a mixture of independent and constrained refinement
*R*[*F*^2^ > 2σ(*F*^2^)] = 0.051	*w* = 1/[σ^2^(*F*_o_^2^) + (0.0621*P*)^2^ + 0.8577*P*] where *P* = (*F*_o_^2^ + 2*F*_c_^2^)/3
*wR*(*F*^2^) = 0.154	(Δ/σ)_max_ < 0.001
*S* = 1.02	Δρ_max_ = 0.48 e Å^−^^3^
4161 reflections	Δρ_min_ = −0.20 e Å^−^^3^
293 parameters	Extinction correction: *SHELXL*, Fc^*^=kFc[1+0.001xFc^2^λ^3^/sin(2θ)]^-1/4^
4 restraints	Extinction coefficient: 0.0040 (10)

### Special details

**Table:**

Geometry. All esds (except the esd in the dihedral angle between two l.s. planes) are estimated using the full covariance matrix. The cell esds are taken into account individually in the estimation of esds in distances, angles and torsion angles; correlations between esds in cell parameters are only used when they are defined by crystal symmetry. An approximate (isotropic) treatment of cell esds is used for estimating esds involving l.s. planes.

### Fractional atomic coordinates and isotropic or equivalent isotropic displacement parameters (Å^2^)

**Table:**

	*x*	*y*	*z*	*U*_iso_*/*U*_eq_	
N1	0.7167 (2)	0.31832 (9)	0.7595 (2)	0.0571 (6)	
H1N	0.736 (3)	0.3010 (10)	0.7008 (18)	0.057 (8)*	
N2	0.7489 (2)	0.37679 (9)	0.97935 (19)	0.0501 (5)	
H2N	0.781 (2)	0.3936 (9)	1.0446 (16)	0.045 (7)*	
C13	0.8453 (2)	0.38831 (9)	0.8995 (2)	0.0428 (6)	
C14	0.8286 (2)	0.36004 (10)	0.7919 (2)	0.0452 (6)	
C15	0.9343 (3)	0.36978 (12)	0.7212 (2)	0.0594 (7)	
H15	0.9214	0.3525	0.6475	0.071*	
C16	1.0578 (3)	0.40420 (12)	0.7569 (3)	0.0630 (7)	
H16	1.1290	0.4089	0.7090	0.076*	
C17	1.0752 (3)	0.43161 (12)	0.8637 (3)	0.0620 (7)	
H17	1.1583	0.4549	0.8891	0.074*	
C18	0.9676 (3)	0.42414 (10)	0.9325 (2)	0.0552 (7)	
H18	0.9774	0.4438	1.0035	0.066*	
C19	0.5856 (3)	0.38711 (10)	0.9469 (2)	0.0545 (7)	
C20	0.5126 (3)	0.33939 (11)	0.8667 (2)	0.0594 (7)	
H20A	0.5330	0.3032	0.9085	0.071*	
H20B	0.4048	0.3453	0.8536	0.071*	
C21	0.5583 (3)	0.33282 (12)	0.7482 (2)	0.0614 (7)	
H21	0.5391	0.3691	0.7047	0.074*	
C22	0.4666 (4)	0.28509 (16)	0.6785 (3)	0.0966 (11)	
H22A	0.4946	0.2817	0.6026	0.145*	
H22B	0.3617	0.2942	0.6689	0.145*	
H22C	0.4858	0.2493	0.7201	0.145*	
C23	0.5232 (4)	0.38475 (14)	1.0604 (3)	0.0832 (9)	
H23A	0.5673	0.4149	1.1122	0.125*	
H23B	0.5467	0.3482	1.0978	0.125*	
H23C	0.4162	0.3897	1.0429	0.125*	
C24	0.5541 (4)	0.44577 (13)	0.8872 (3)	0.0859 (10)	
H24A	0.5998	0.4755	0.9394	0.129*	
H24B	0.4477	0.4520	0.8684	0.129*	
H24C	0.5952	0.4466	0.8163	0.129*	
N3	0.9623 (2)	0.08032 (10)	0.75961 (19)	0.0552 (6)	
H3N	0.959 (3)	0.0442 (8)	0.738 (2)	0.060 (8)*	
N4	0.9115 (2)	0.19484 (10)	0.8412 (2)	0.0564 (6)	
H4N	0.878 (3)	0.2259 (9)	0.867 (2)	0.071 (9)*	
C1	0.8201 (3)	0.14813 (10)	0.8611 (2)	0.0485 (6)	
C2	0.6969 (3)	0.15719 (13)	0.9136 (2)	0.0608 (7)	
H2	0.6789	0.1940	0.9392	0.073*	
C3	0.6004 (3)	0.11377 (14)	0.9293 (3)	0.0703 (8)	
H3	0.5172	0.1214	0.9636	0.084*	
C4	0.6268 (3)	0.05903 (14)	0.8944 (3)	0.0698 (8)	
H4	0.5626	0.0291	0.9056	0.084*	
C5	0.7493 (3)	0.04893 (11)	0.8427 (2)	0.0600 (7)	
H5	0.7674	0.0117	0.8195	0.072*	
C6	0.8469 (3)	0.09240 (11)	0.8238 (2)	0.0480 (6)	
C7	1.1215 (3)	0.09048 (12)	0.8137 (2)	0.0566 (7)	
C8	1.1544 (3)	0.15471 (13)	0.8175 (3)	0.0656 (8)	
H8A	1.2614	0.1596	0.8452	0.079*	
H8B	1.1324	0.1690	0.7374	0.079*	
C9	1.0732 (3)	0.19262 (11)	0.8915 (3)	0.0605 (7)	
H9	1.0863	0.1767	0.9713	0.073*	
C10	1.2146 (3)	0.06159 (16)	0.7333 (3)	0.0893 (11)	
H10A	1.1937	0.0211	0.7296	0.134*	
H10B	1.3194	0.0676	0.7636	0.134*	
H10C	1.1896	0.0778	0.6558	0.134*	
C11	1.1571 (3)	0.06352 (13)	0.9354 (3)	0.0776 (9)	
H11A	1.1452	0.0225	0.9288	0.116*	
H11B	1.0899	0.0786	0.9833	0.116*	
H11C	1.2588	0.0724	0.9710	0.116*	
C12	1.1335 (3)	0.25374 (14)	0.8980 (3)	0.0984 (12)	
H12A	1.0794	0.2768	0.9452	0.148*	
H12B	1.1209	0.2696	0.8201	0.148*	
H12C	1.2382	0.2535	0.9327	0.148*	
O1	0.8427 (3)	0.26647 (10)	1.0918 (3)	0.0817 (7)	
H1W	0.861 (4)	0.2794 (15)	1.161 (3)	0.098 (14)*	
H2W	0.809 (4)	0.2957 (17)	1.050 (3)	0.114 (15)*	

### Atomic displacement parameters (Å^2^)

**Table:**

	*U*^11^	*U*^22^	*U*^33^	*U*^12^	*U*^13^	*U*^23^
N1	0.0624 (15)	0.0558 (13)	0.0550 (15)	−0.0084 (11)	0.0155 (12)	−0.0159 (11)
N2	0.0565 (13)	0.0543 (13)	0.0407 (13)	−0.0018 (10)	0.0117 (11)	−0.0061 (11)
C13	0.0454 (14)	0.0404 (13)	0.0432 (15)	0.0044 (11)	0.0098 (11)	0.0019 (11)
C14	0.0455 (14)	0.0449 (14)	0.0450 (15)	0.0016 (11)	0.0078 (11)	0.0002 (11)
C15	0.0599 (17)	0.0710 (18)	0.0489 (16)	−0.0039 (14)	0.0142 (13)	−0.0050 (13)
C16	0.0556 (17)	0.0743 (18)	0.0628 (19)	−0.0033 (14)	0.0206 (14)	0.0047 (16)
C17	0.0502 (16)	0.0662 (18)	0.068 (2)	−0.0099 (13)	0.0071 (14)	0.0016 (15)
C18	0.0579 (16)	0.0551 (16)	0.0511 (17)	−0.0044 (13)	0.0058 (13)	−0.0073 (12)
C19	0.0508 (15)	0.0552 (16)	0.0618 (18)	0.0055 (12)	0.0216 (13)	0.0000 (13)
C20	0.0494 (15)	0.0621 (17)	0.0684 (19)	−0.0023 (12)	0.0150 (13)	0.0008 (14)
C21	0.0542 (16)	0.0690 (18)	0.0604 (19)	−0.0100 (13)	0.0086 (13)	−0.0041 (14)
C22	0.080 (2)	0.120 (3)	0.088 (3)	−0.040 (2)	0.0105 (18)	−0.031 (2)
C23	0.082 (2)	0.097 (2)	0.080 (2)	−0.0006 (18)	0.0405 (18)	−0.0111 (18)
C24	0.079 (2)	0.069 (2)	0.113 (3)	0.0140 (16)	0.0257 (19)	0.0008 (18)
N3	0.0463 (13)	0.0679 (16)	0.0517 (14)	0.0055 (11)	0.0100 (10)	−0.0027 (12)
N4	0.0496 (13)	0.0521 (14)	0.0662 (16)	0.0015 (11)	0.0073 (11)	0.0007 (11)
C1	0.0411 (14)	0.0554 (16)	0.0462 (15)	0.0010 (12)	0.0006 (11)	0.0032 (12)
C2	0.0429 (15)	0.0755 (19)	0.0633 (18)	0.0046 (14)	0.0081 (13)	−0.0101 (14)
C3	0.0455 (16)	0.095 (2)	0.073 (2)	0.0023 (16)	0.0178 (14)	−0.0012 (17)
C4	0.0516 (17)	0.080 (2)	0.079 (2)	−0.0084 (15)	0.0143 (15)	0.0148 (17)
C5	0.0556 (16)	0.0565 (16)	0.0675 (19)	0.0021 (13)	0.0100 (14)	0.0074 (13)
C6	0.0399 (14)	0.0614 (16)	0.0418 (15)	0.0022 (12)	0.0052 (11)	0.0058 (12)
C7	0.0438 (15)	0.0738 (19)	0.0522 (17)	0.0099 (13)	0.0087 (12)	0.0064 (14)
C8	0.0404 (14)	0.090 (2)	0.0657 (19)	−0.0016 (14)	0.0076 (13)	0.0205 (16)
C9	0.0480 (15)	0.0640 (18)	0.0665 (19)	−0.0068 (13)	0.0027 (13)	0.0078 (14)
C10	0.0529 (18)	0.131 (3)	0.086 (2)	0.0169 (18)	0.0190 (16)	−0.014 (2)
C11	0.0698 (19)	0.084 (2)	0.075 (2)	0.0150 (15)	0.0010 (16)	0.0181 (17)
C12	0.069 (2)	0.074 (2)	0.145 (3)	−0.0189 (17)	0.001 (2)	0.008 (2)
O1	0.120 (2)	0.0592 (14)	0.0643 (16)	−0.0014 (13)	0.0126 (14)	0.0038 (13)

### Geometric parameters (Å, º)

**Table:**

N1—C14	1.404 (3)	N3—C7	1.482 (3)
N1—C21	1.455 (3)	N3—H3N	0.874 (16)
N1—H1N	0.835 (16)	N4—C1	1.410 (3)
N2—C13	1.406 (3)	N4—C9	1.475 (3)
N2—C19	1.477 (3)	N4—H4N	0.856 (17)
N2—H2N	0.852 (16)	C1—C2	1.380 (3)
C13—C18	1.382 (3)	C1—C6	1.400 (3)
C13—C14	1.391 (3)	C2—C3	1.368 (4)
C14—C15	1.386 (3)	C2—H2	0.9300
C15—C16	1.376 (4)	C3—C4	1.369 (4)
C15—H15	0.9300	C3—H3	0.9300
C16—C17	1.372 (4)	C4—C5	1.373 (4)
C16—H16	0.9300	C4—H4	0.9300
C17—C18	1.377 (4)	C5—C6	1.386 (3)
C17—H17	0.9300	C5—H5	0.9300
C18—H18	0.9300	C7—C10	1.520 (4)
C19—C20	1.518 (4)	C7—C11	1.520 (4)
C19—C23	1.521 (4)	C7—C8	1.522 (4)
C19—C24	1.532 (4)	C8—C9	1.510 (4)
C20—C21	1.510 (4)	C8—H8A	0.9700
C20—H20A	0.9700	C8—H8B	0.9700
C20—H20B	0.9700	C9—C12	1.519 (4)
C21—C22	1.525 (4)	C9—H9	0.9800
C21—H21	0.9800	C10—H10A	0.9600
C22—H22A	0.9600	C10—H10B	0.9600
C22—H22B	0.9600	C10—H10C	0.9600
C22—H22C	0.9600	C11—H11A	0.9600
C23—H23A	0.9600	C11—H11B	0.9600
C23—H23B	0.9600	C11—H11C	0.9600
C23—H23C	0.9600	C12—H12A	0.9600
C24—H24A	0.9600	C12—H12B	0.9600
C24—H24B	0.9600	C12—H12C	0.9600
C24—H24C	0.9600	O1—H1W	0.84 (4)
N3—C6	1.415 (3)	O1—H2W	0.86 (4)
			
C14—N1—C21	121.0 (2)	C6—N3—H3N	110.3 (17)
C14—N1—H1N	107.4 (18)	C7—N3—H3N	104.9 (17)
C21—N1—H1N	113.0 (18)	C1—N4—C9	118.6 (2)
C13—N2—C19	121.0 (2)	C1—N4—H4N	109.9 (18)
C13—N2—H2N	109.9 (16)	C9—N4—H4N	106.1 (19)
C19—N2—H2N	108.4 (15)	C2—C1—C6	118.4 (2)
C18—C13—C14	118.9 (2)	C2—C1—N4	120.0 (2)
C18—C13—N2	119.7 (2)	C6—C1—N4	121.5 (2)
C14—C13—N2	121.2 (2)	C3—C2—C1	122.2 (3)
C15—C14—C13	118.4 (2)	C3—C2—H2	118.9
C15—C14—N1	119.9 (2)	C1—C2—H2	118.9
C13—C14—N1	121.4 (2)	C2—C3—C4	119.8 (3)
C16—C15—C14	121.9 (3)	C2—C3—H3	120.1
C16—C15—H15	119.0	C4—C3—H3	120.1
C14—C15—H15	119.0	C3—C4—C5	119.0 (3)
C17—C16—C15	119.6 (3)	C3—C4—H4	120.5
C17—C16—H16	120.2	C5—C4—H4	120.5
C15—C16—H16	120.2	C4—C5—C6	122.2 (3)
C16—C17—C18	118.9 (3)	C4—C5—H5	118.9
C16—C17—H17	120.5	C6—C5—H5	118.9
C18—C17—H17	120.5	C5—C6—C1	118.3 (2)
C17—C18—C13	122.1 (2)	C5—C6—N3	119.4 (2)
C17—C18—H18	118.9	C1—C6—N3	122.1 (2)
C13—C18—H18	118.9	N3—C7—C10	106.1 (2)
N2—C19—C20	109.9 (2)	N3—C7—C11	110.6 (2)
N2—C19—C23	106.7 (2)	C10—C7—C11	109.6 (2)
C20—C19—C23	108.2 (2)	N3—C7—C8	109.8 (2)
N2—C19—C24	110.8 (2)	C10—C7—C8	108.9 (2)
C20—C19—C24	110.5 (2)	C11—C7—C8	111.7 (2)
C23—C19—C24	110.7 (2)	C9—C8—C7	118.4 (2)
C21—C20—C19	117.9 (2)	C9—C8—H8A	107.7
C21—C20—H20A	107.8	C7—C8—H8A	107.7
C19—C20—H20A	107.8	C9—C8—H8B	107.7
C21—C20—H20B	107.8	C7—C8—H8B	107.7
C19—C20—H20B	107.8	H8A—C8—H8B	107.1
H20A—C20—H20B	107.2	N4—C9—C8	110.3 (2)
N1—C21—C20	111.7 (2)	N4—C9—C12	108.0 (2)
N1—C21—C22	108.2 (2)	C8—C9—C12	111.5 (2)
C20—C21—C22	109.9 (2)	N4—C9—H9	109.0
N1—C21—H21	109.0	C8—C9—H9	109.0
C20—C21—H21	109.0	C12—C9—H9	109.0
C22—C21—H21	109.0	C7—C10—H10A	109.5
C21—C22—H22A	109.5	C7—C10—H10B	109.5
C21—C22—H22B	109.5	H10A—C10—H10B	109.5
H22A—C22—H22B	109.5	C7—C10—H10C	109.5
C21—C22—H22C	109.5	H10A—C10—H10C	109.5
H22A—C22—H22C	109.5	H10B—C10—H10C	109.5
H22B—C22—H22C	109.5	C7—C11—H11A	109.5
C19—C23—H23A	109.5	C7—C11—H11B	109.5
C19—C23—H23B	109.5	H11A—C11—H11B	109.5
H23A—C23—H23B	109.5	C7—C11—H11C	109.5
C19—C23—H23C	109.5	H11A—C11—H11C	109.5
H23A—C23—H23C	109.5	H11B—C11—H11C	109.5
H23B—C23—H23C	109.5	C9—C12—H12A	109.5
C19—C24—H24A	109.5	C9—C12—H12B	109.5
C19—C24—H24B	109.5	H12A—C12—H12B	109.5
H24A—C24—H24B	109.5	C9—C12—H12C	109.5
C19—C24—H24C	109.5	H12A—C12—H12C	109.5
H24A—C24—H24C	109.5	H12B—C12—H12C	109.5
H24B—C24—H24C	109.5	H1W—O1—H2W	104 (3)
C6—N3—C7	120.1 (2)		
			
C19—N2—C13—C18	125.4 (2)	C9—N4—C1—C2	123.2 (3)
C19—N2—C13—C14	−60.9 (3)	C9—N4—C1—C6	−59.8 (3)
C18—C13—C14—C15	−1.1 (3)	C6—C1—C2—C3	−0.5 (4)
N2—C13—C14—C15	−174.8 (2)	N4—C1—C2—C3	176.6 (2)
C18—C13—C14—N1	172.8 (2)	C1—C2—C3—C4	1.3 (4)
N2—C13—C14—N1	−1.0 (3)	C2—C3—C4—C5	−0.8 (4)
C21—N1—C14—C15	−125.1 (3)	C3—C4—C5—C6	−0.4 (4)
C21—N1—C14—C13	61.1 (3)	C4—C5—C6—C1	1.2 (4)
C13—C14—C15—C16	3.1 (4)	C4—C5—C6—N3	−173.7 (2)
N1—C14—C15—C16	−170.8 (2)	C2—C1—C6—C5	−0.7 (3)
C14—C15—C16—C17	−2.4 (4)	N4—C1—C6—C5	−177.8 (2)
C15—C16—C17—C18	−0.3 (4)	C2—C1—C6—N3	174.1 (2)
C16—C17—C18—C13	2.3 (4)	N4—C1—C6—N3	−3.0 (4)
C14—C13—C18—C17	−1.6 (4)	C7—N3—C6—C5	−122.3 (3)
N2—C13—C18—C17	172.2 (2)	C7—N3—C6—C1	63.0 (3)
C13—N2—C19—C20	76.8 (3)	C6—N3—C7—C10	168.2 (2)
C13—N2—C19—C23	−166.1 (2)	C6—N3—C7—C11	49.4 (3)
C13—N2—C19—C24	−45.6 (3)	C6—N3—C7—C8	−74.3 (3)
N2—C19—C20—C21	−63.4 (3)	N3—C7—C8—C9	63.8 (3)
C23—C19—C20—C21	−179.5 (2)	C10—C7—C8—C9	179.5 (2)
C24—C19—C20—C21	59.2 (3)	C11—C7—C8—C9	−59.3 (3)
C14—N1—C21—C20	−75.5 (3)	C1—N4—C9—C8	78.6 (3)
C14—N1—C21—C22	163.4 (2)	C1—N4—C9—C12	−159.3 (3)
C19—C20—C21—N1	63.1 (3)	C7—C8—C9—N4	−67.3 (3)
C19—C20—C21—C22	−176.8 (2)	C7—C8—C9—C12	172.7 (2)

### Hydrogen-bond geometry (Å, º)

**Table:**

*D*—H···*A*	*D*—H	H···*A*	*D*···*A*	*D*—H···*A*
N1—H1*N*···O1^i^	0.84 (2)	2.33 (2)	3.122 (3)	159 (2)
O1—H1*W*···N4^ii^	0.84 (4)	2.14 (4)	2.976 (4)	175 (3)
O1—H2*W*···N2	0.86 (4)	2.09 (4)	2.930 (3)	167 (4)

Symmetry codes: (i) *x*, −*y*+1/2, *z*−1/2; (ii) *x*, −*y*+1/2, *z*+1/2.
