# Supplementary figures and images for: Crystal structure of 2,2,4-trimethyl-2,3,4,5-tetra­hydro-1H-benzo[b][1,4]diazepine hemihydrate
Source: Acta Crystallogr E Crystallogr Commun. 2015 Jul 15;71(Pt 8):o570–1. doi: 10.1107/S2056989015013201 (PMC4571402; doi:10.1107/S2056989015013201)

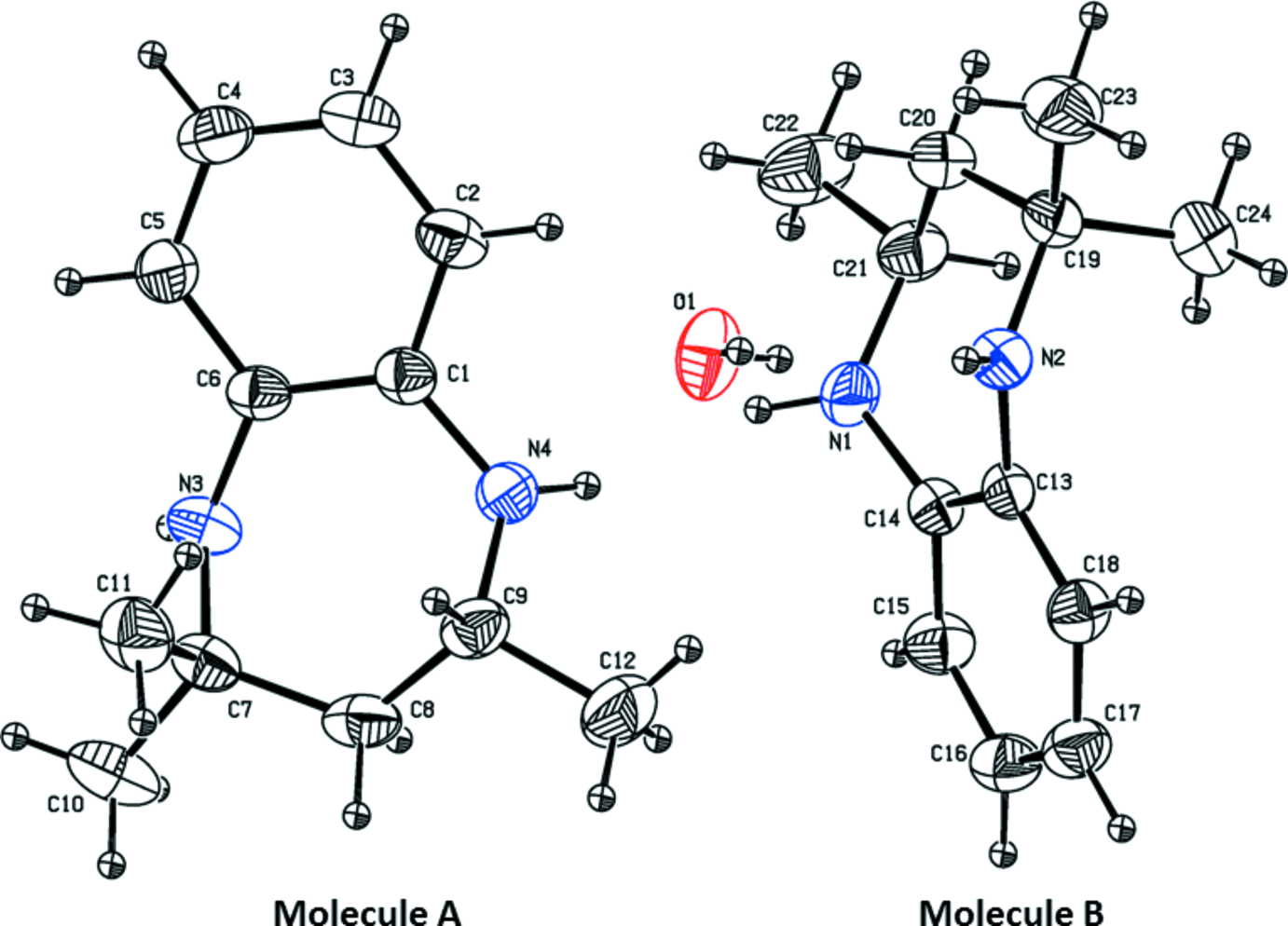

Supplement: Supplementary file 4 [file e-71-0o570-fig1.tif]

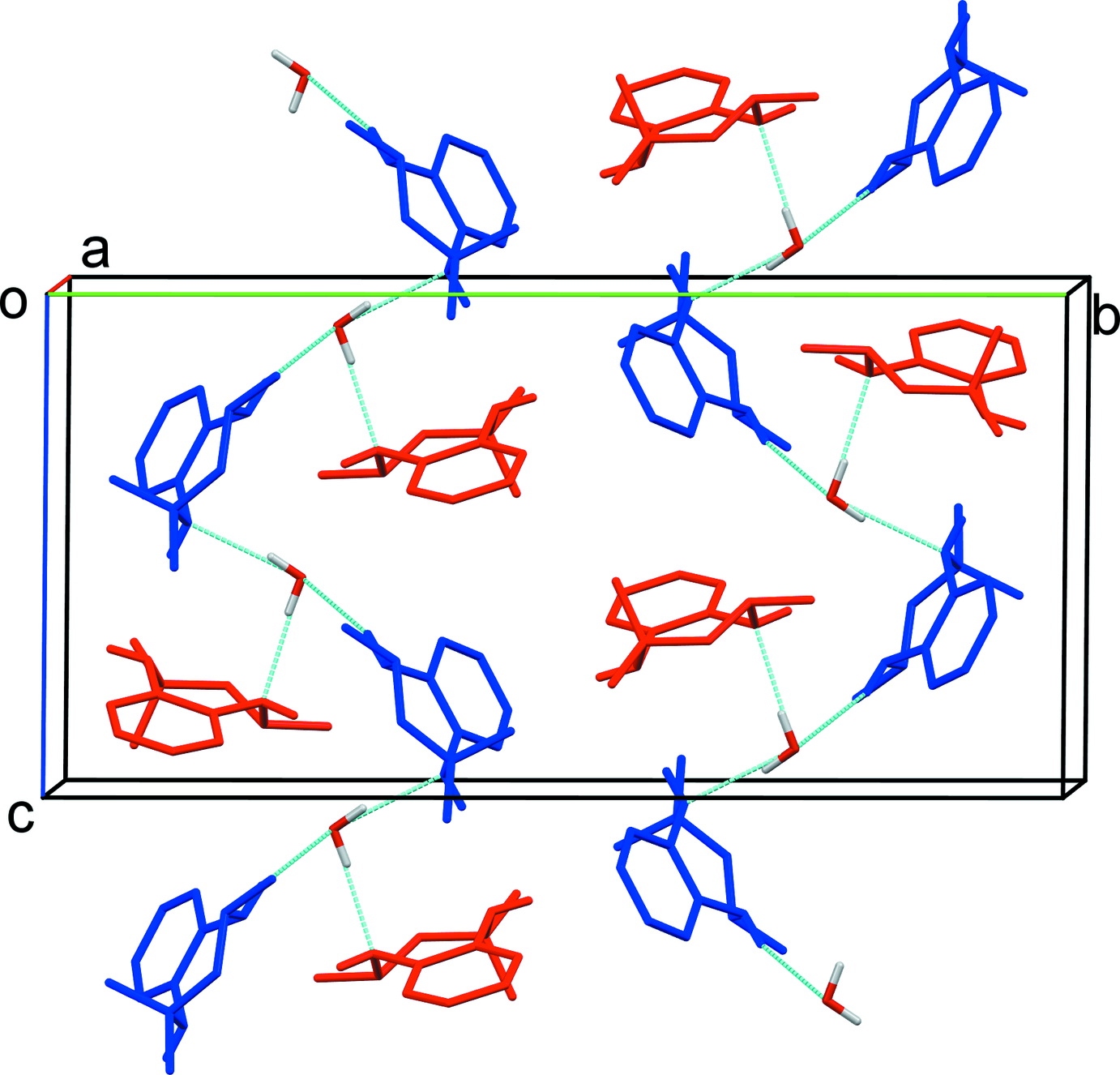

Supplement: Supplementary file 5 [file e-71-0o570-fig2.tif]
